# Supplementary material for: Interaction Study between ESIPT Fluorescent Lipophile-Based Benzazoles and BSA
Source: Molecules. 2021 Nov 6;26(21):6728. doi: 10.3390/molecules26216728 (PMC8586955; doi:10.3390/molecules26216728)
Supplement: Supplementary file 1 [file molecules-26-06728-s001.zip › molecules-1434793-supplementary.pdf]

Interaction study between ESIPT fluorescent lipophile-based benzazoles and BSA

Thais Kroetz,[1] Pablo Andrei Nogara,[2] Fabiano da Silveira Santos,[1,3] Lilian Camargo da Luz,[1] Viktor Saraiva Câmara,[1] João Batista Teixeira da Rocha,[2] Alexandre Gonçalves Dal-Bó,[3] Fabiano Severo Rodembusch,[1,*]

[1]Grupo de Pesquisa em Fotoquímica Orgânica Aplicada, Instituto de Química, Universidade Federal do Rio Grande do Sul, Av. Bento Gonçalves 9500, Porto Alegre, RS, Brazil; e-mails: kroetzthais@gmail.com (T.K.); lilian.luz@ufrgs.br (L.C.L.); fabiano@ufrgs.br (F.S.S.); viktor.saraiva@gmail.com (V.S.C.); rodembusch@iq.ufrgs.br (F.S.R.)

[2]Departamento de Bioquímica e Biologia Molecular, Centro de Ciências Naturais e Exatas, Universidade Federal de Santa Maria - UFSM, RS, Brazil; e-mails: pbnogara@gmail.com (P.A.N.); jbtrocha@yahoo.com.br (J.B.T.R.)

[3]Universidade do Extremo Sul Catarinense – UNESC, Av. Universitária 1105, CEP 88806-000, Criciúma, SC, Brazil; e-mails: fabiano@ufrgs.br (F.S.S.); adalbo@unesc.net (A.G.D.)

*Author to whom correspondence should be addressed; e-mail: rodembusch@iq.ufrgs.br (F.S.R.); Tel.: +55 51 3308 9639; Fax: +55 51 3308 7304.

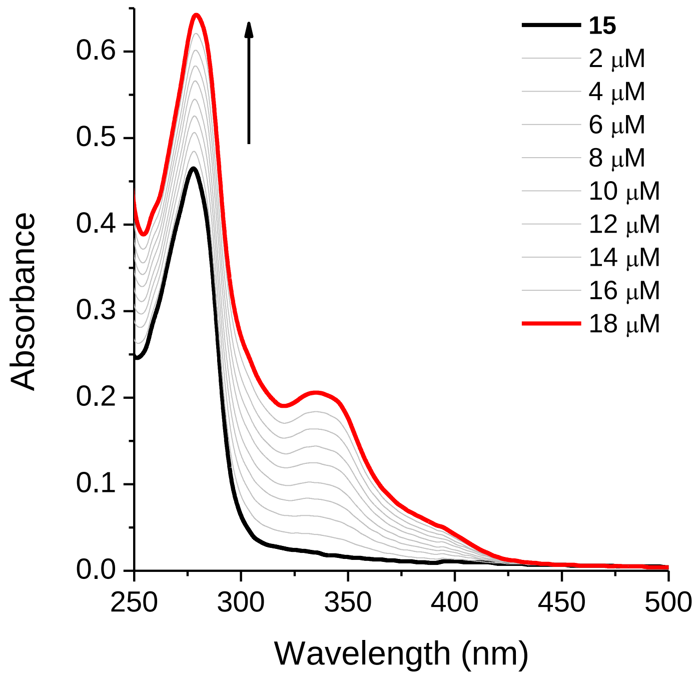

Figure S1. UV-Vis absorption spectra for BSA (11 μM) in PBS (pH 7.2) in the absence and presence of different amounts of compound **15** (0 to 20 μM).

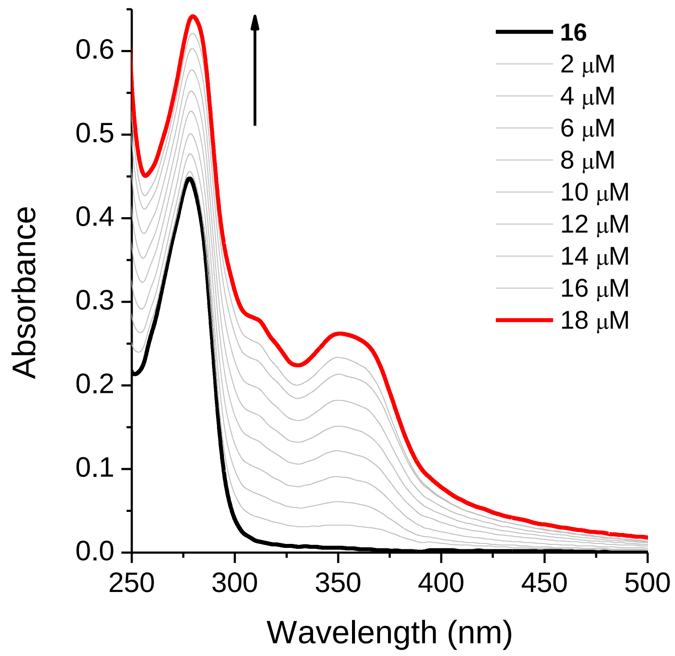

Figure S2. UV-Vis absorption spectra for BSA (11 μM) in PBS (pH 7.2) in the absence and presence of different amounts of compound **16** (0 to 20 μM).

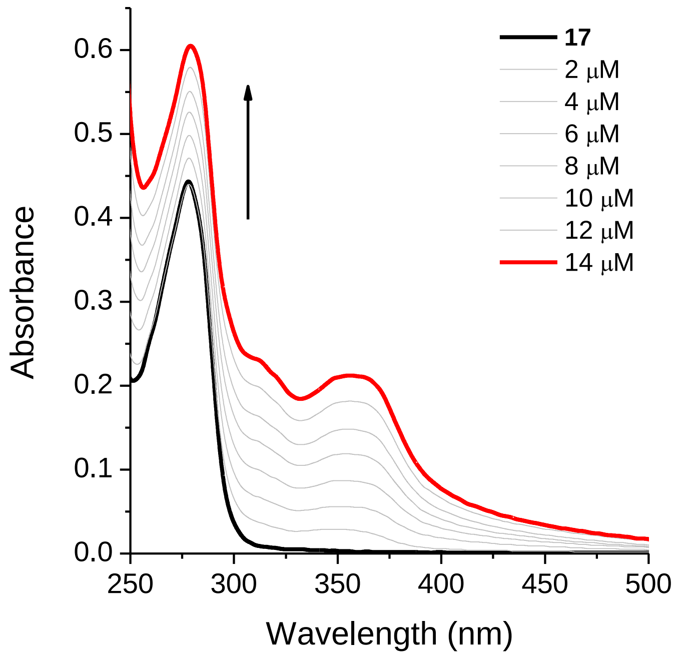

Figure S3. UV-Vis absorption spectra for BSA (11 μM) in PBS (pH 7.2) in the absence and presence of different amounts of compound **17** (0 to 20 μM).

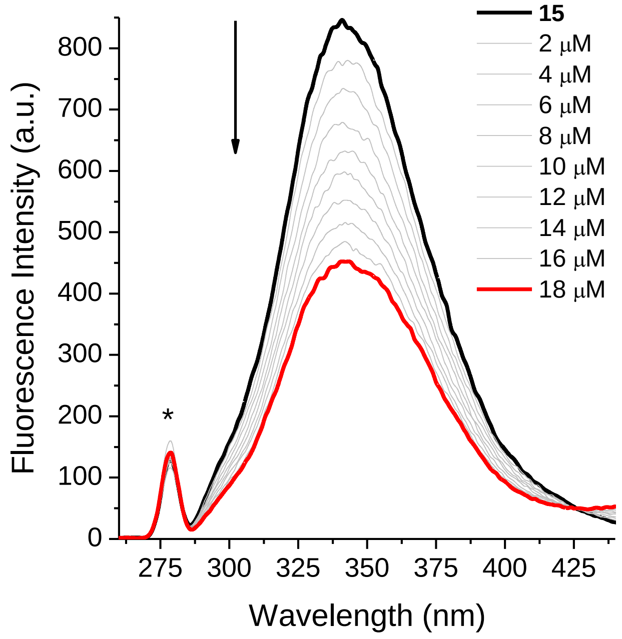

Figure S4. Steady-state fluorescence emission spectra for BSA (11 μM) in PBS (pH 7.2) in the absence and presence of different amounts of compound **15** (0 to 20 μM). The asterisk is related to the excitation wavelength. (λ_{ex} = 277 nm, Slits (Ex/Em) = 5.0 nm/5.0 nm)

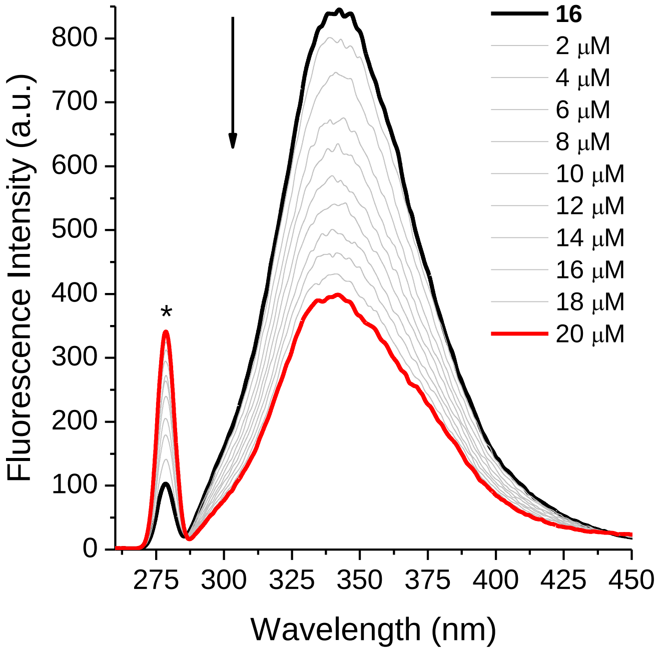

Figure S5. Steady-state fluorescence emission spectra for BSA (11 μM) in PBS (pH 7.2) in the absence and presence of different amounts of compound **16** (0 to 20 μM). The asterisk is related to the excitation wavelength. (λ_{ex} = 277 nm, Slits (Ex/Em) = 5.0 nm/5.0 nm)

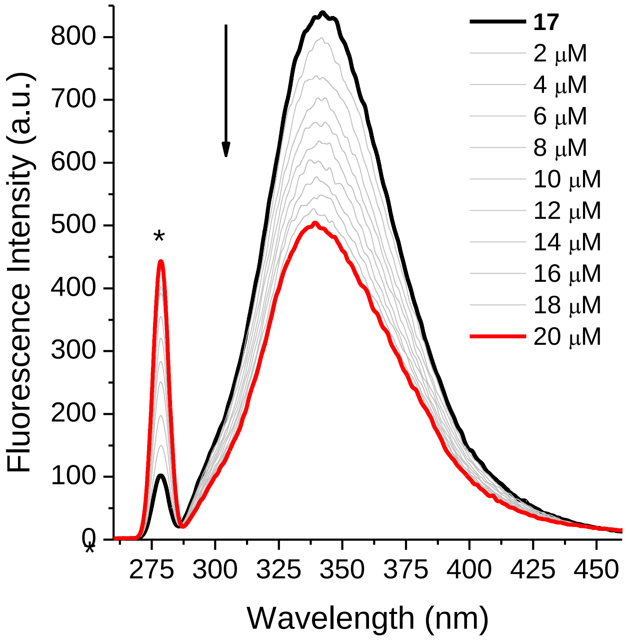

Figure S6. Steady-state fluorescence emission spectra for BSA (11 μM) in PBS (pH 7.2) in the absence and presence of different amounts of compound **17** (0 to 20 μM). The asterisk is related to the excitation wavelength. (λ_{ex} = 277 nm, Slits (Ex/Em) = 5.0 nm/5.0 nm)

Table S1. Linear equations and respective coefficient of determination (R^2) from the plot F_0/F *vs*. [Q] (Stern-Volmer relation) and log [(F_0-F)/F] *vs*. log [Q] (double log plot).

**Benzazole**	**Stern-Volmer relation**		**Double log plot**	
	**Equation**	**R^2**	**Equation**	**R^2**
**12**	$F_0/F=3.14x10^4$ [Q]+1.0022	0.9969	log(F_0-F/F)=4.10+0.92 log [Q]	0.9928
**13**	$F_0/F=5.12x10^4$ [Q]+0.9746	0.9986	log(F_0-F/F)=5.50+1.16 log [Q]	0.9953
**14**	$F_0/F=3.18x10^4$ [Q]+1.0180	0.9927	log(F_0-F/F)=3.91+0.88 log [Q]	0.9878
**15**	$F_0/F=4.66x10^4$ [Q]+0.9663	0.9916	log(F_0-F/F)=5.35+1.15 log [Q]	0.9983
**16**	$F_0/F=5.62x10^4$ [Q]+0.9237	0.9859	log(F_0-F/F)=6.18+1.31 log [Q]	0.9989
**17**	$F_0/F=3.46x10^4$ [Q]+0.9890	0.9975	log(F_0-F/F)=4.95+1.09 log [Q]	0.9933

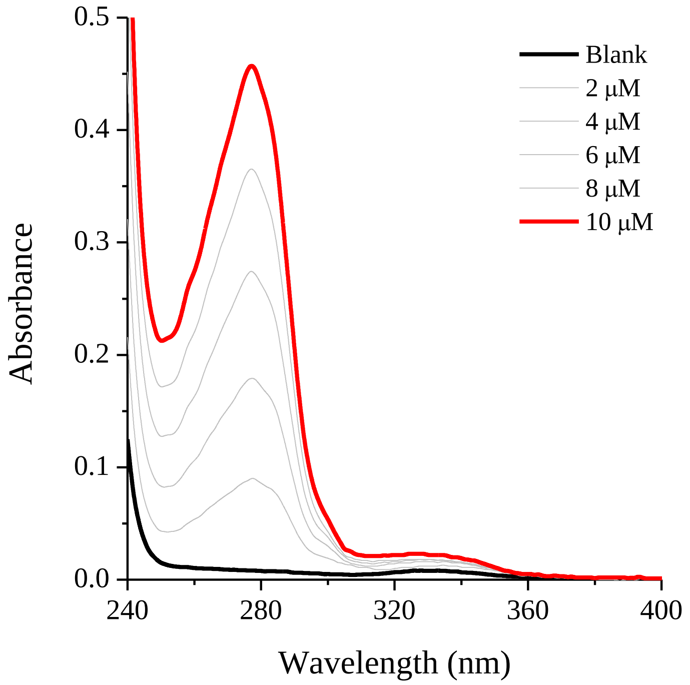

Figure S7. UV-Vis spectra of the benzazole 13 [2 μM] in different BSA concentrations [0-10 μM]. Blank sample is the UV-VIs in absence of BSA.

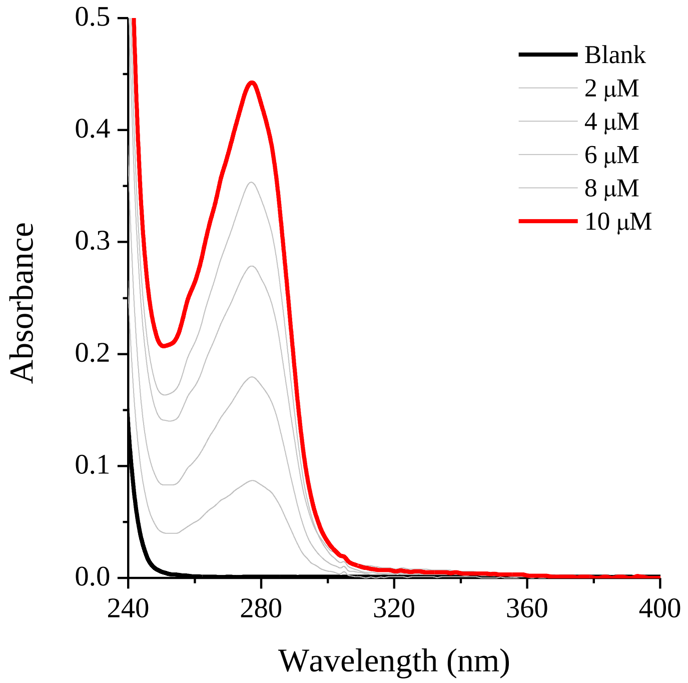

Figure S8. UV-Vis spectra of the benzazole 14 [2 μM] in different BSA concentrations [0-10 μM]. Blank sample is the UV-VIs in absence of BSA.

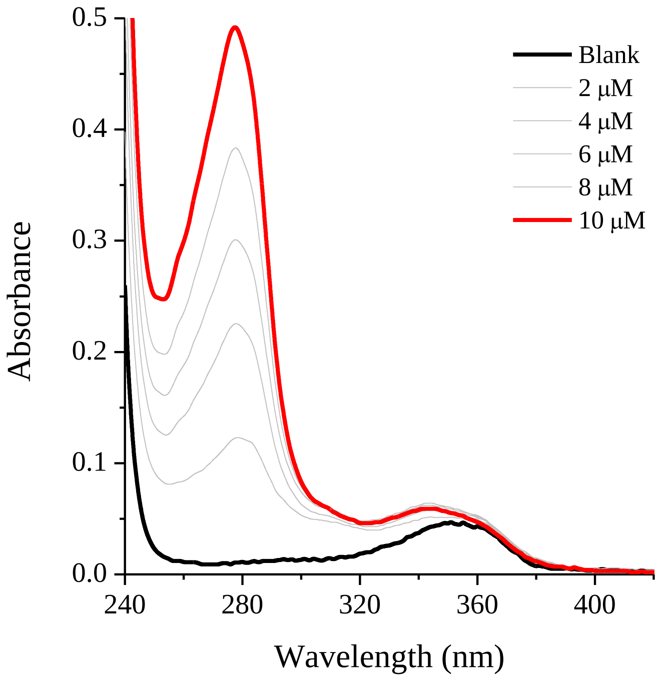

Figure S9. UV-Vis spectra of the benzazole 16 [2 μM] in different BSA concentrations [0-10 μM]. Blank sample is the UV-VIs in absence of BSA.

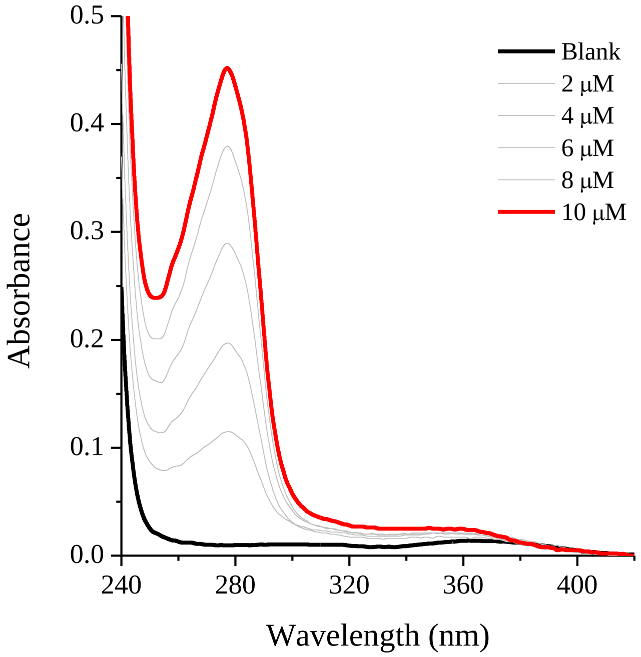

Figure S10. UV-Vis spectra of the benzazole 17 [2 μM] in different BSA concentrations [0-10 μM]. Blank sample is the UV-VIs in absence of BSA.

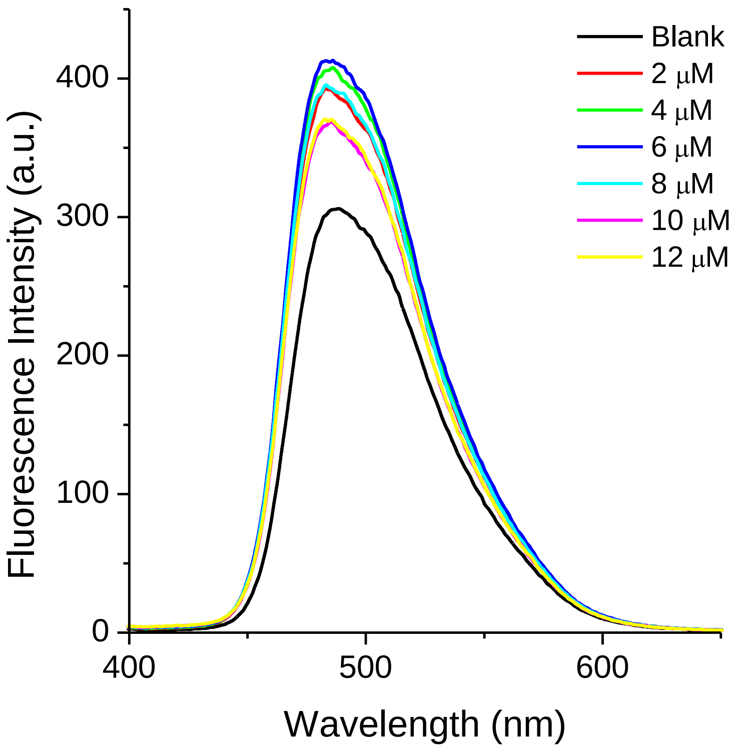

Figure S11. Fluorescence emission spectra of the benzazole 13 [2 μM] in different BSA concentrations [0-10 μM]. Blank sample is the UV-VIs in absence of BSA.

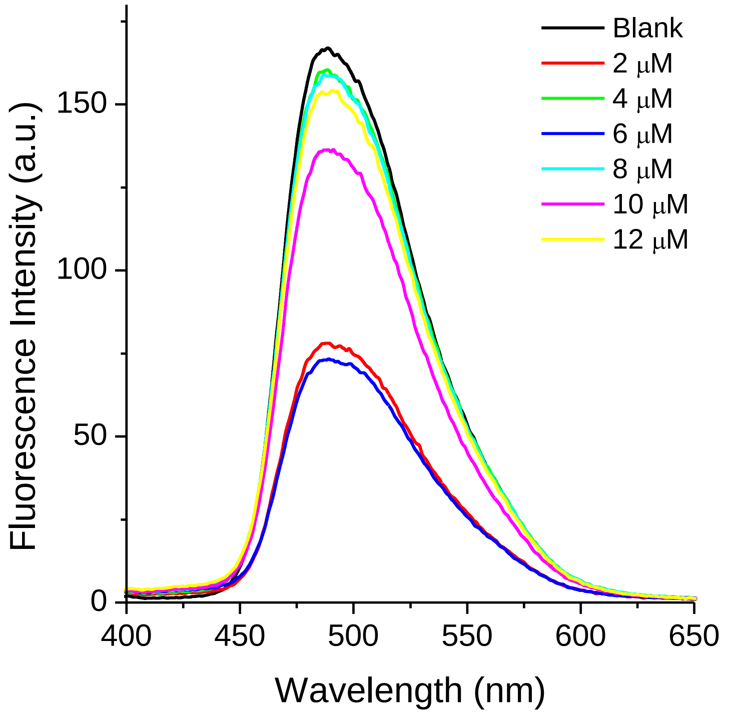

Figure S12. Fluorescence emission spectra of the benzazole 14 [2 μM] in different BSA concentrations [0-10 μM]. Blank sample is the UV-VIs in absence of BSA.

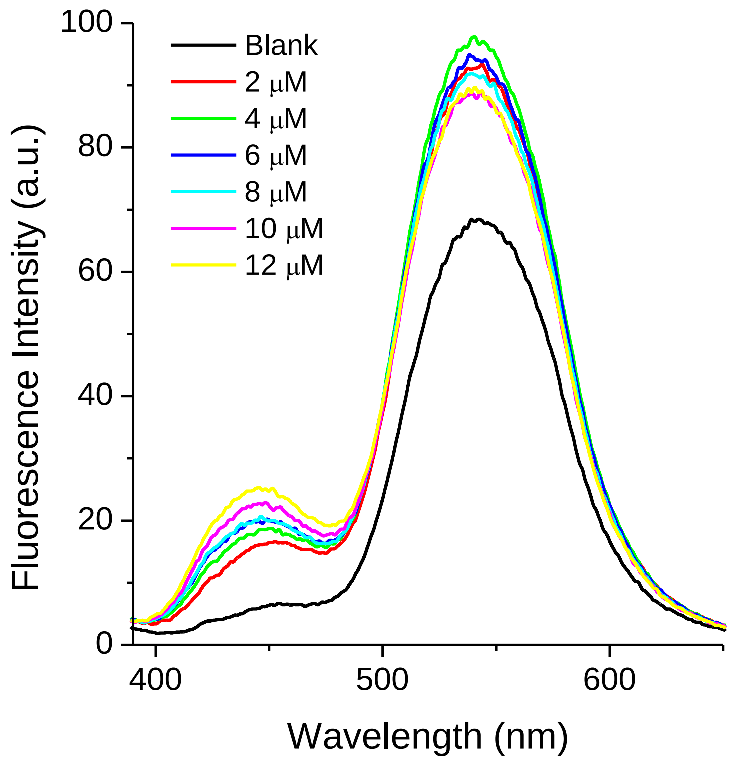

Figure S13. Fluorescence emission spectra of the benzazole 16 [2 μM] in different BSA concentrations [0-10 μM]. Blank sample is the UV-VIs in absence of BSA.

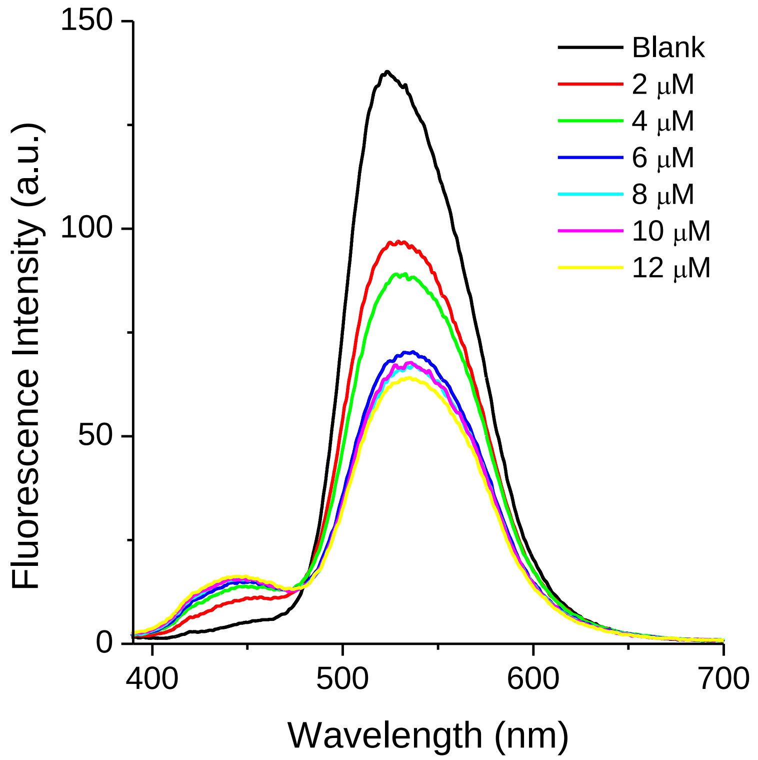

Figure S14. Fluorescence emission spectra of the benzazole 17 [2 μM] in different BSA concentrations [0-10 μM]. Blank sample is the UV-VIs in absence of BSA.

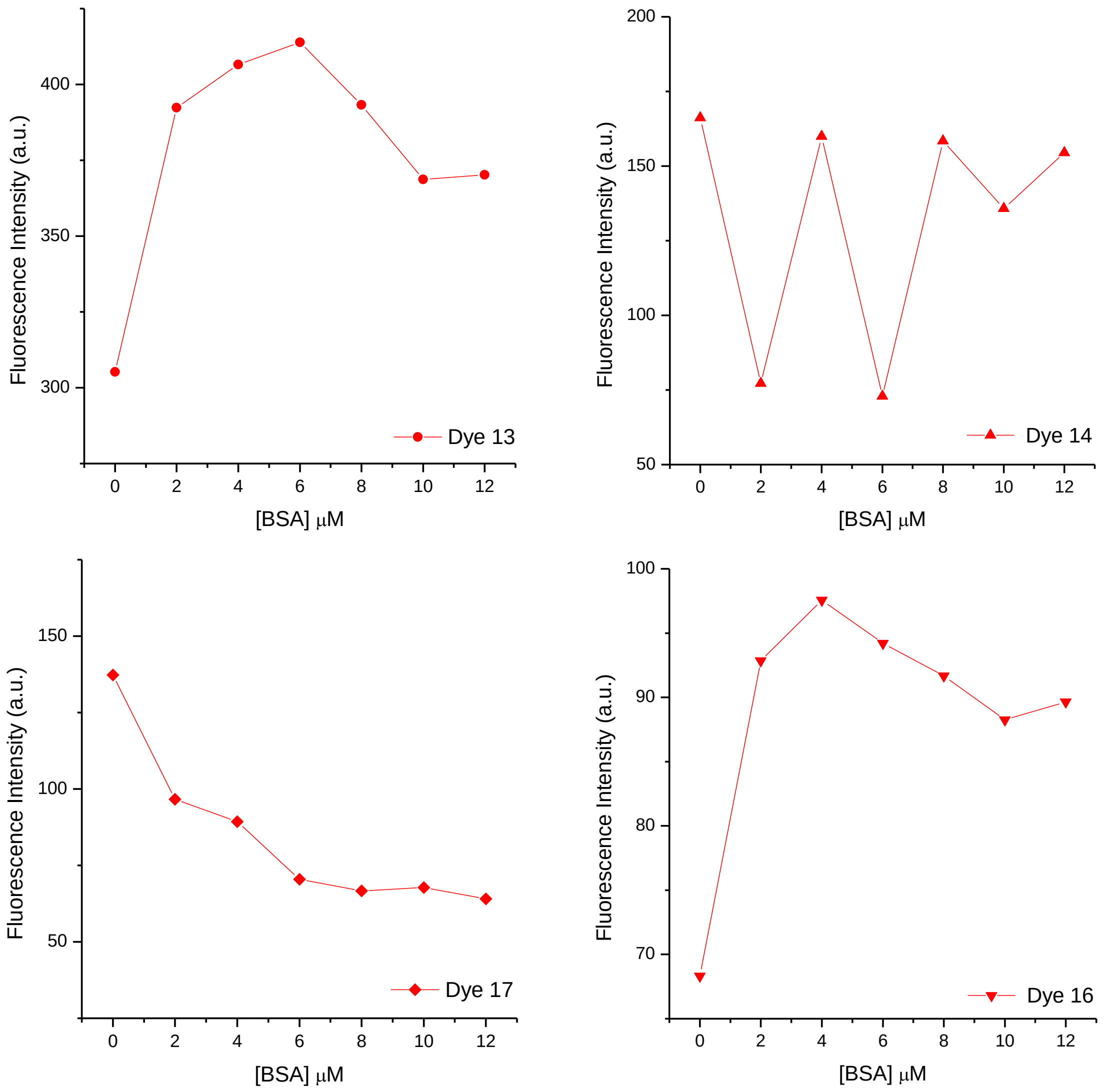

Figure S15. Plot of fluorescence intensity maxima as a function of BSA concentration.
